# Supplementary material for: Protective Effects of Polyphenol-Rich Extracts against Neurotoxicity Elicited by Paraquat or Rotenone in Cellular Models of Parkinson’s Disease
Source: Antioxidants (Basel). 2023 Jul 20;12(7):1463. doi: 10.3390/antiox12071463 (PMC10376534; doi:10.3390/antiox12071463)
Supplement: Supplementary file 1 [file antioxidants-12-01463-s001.zip › antioxidants-2391188-supplementary.pdf]

Supplementary Table S1. Anthocyanin composition of wild BB extract

Anthocyanin	Concentration (mg/g)
1. Delphinidin-3-galactoside	2.79
2. Delphinidin-3-glucoside	3.28
3. Cyanidin-3-galactoside	1.43
4. Delphinidin-3-arabinoside	1.91
5. Cyanidin-3-glucoside	1.76
6. Petunidin-3-galactoside	1.49
7. Cyanidin-3-arabinoside	1.11
8. Petunidin-3-glucoside	2.5
9. Peonidin-3-galactoside	0.7
10. Petunidin-3-arabinoside	0.95
11. Malvidin-3-galactoside	2.9
12. Malvidin-3-glucoside	4.22
13. Malvidin-3-arabinoside	1.76
14. Delphinidin-6-acetyl-3-glucoside	1.19
15. Cyanidin-6-acetyl-3-glucoside	0.53
16. Malvidin-6-acetyl-3-galactoside	0.77
17. Petunidin-6-acetyl-glucoside	0.89
18. Malvidin-6-acetyl-3-glucoside	1.94
Total ANC	29.65

Supplementary Table S2. Anthocyanin composition of BB, BC, and plum extracts[a,b]

	BB	BC	plum
**Anthocyanin[c]**		**Concentration (mg/g)**	
1. Delphinidin-3-O-galactoside	18.8	-	-
2. Delphinidin-3-O-glucoside	9.37	50.4	-
3. Cyanidin-3-O-galactoside	6.23	-	-
4. Delphinidin-3-O-arabinoside	11.3	-	-
5. Delphinidin-3-O-rutinoside	-	153	-
6. Cyanidin-3-O-glucoside	2.96	25.8	14.3
7. Cyanidin-3-O-arabinoside	15.9	-	-
8. Cyanidin-3-O-rutinoside	-	153	10.8
9. Petunidin-3-O-glucoside	9.67	-	-
10. Peonidin-3-O-galactoside	2.49	-	-
11. Petunidin-3-O-arabinoside	6.58	-	-
12. Malvidin-3-O-galactoside	36.3	-	-
13. Malvidin-3-O-glucoside	26.5	-	-
14. Malvidin-3-O-arabinoside	21.3	-	-
15. Delphinidin-3-O-(6''-acetyl-glucoside)	6.05	-	-
16. Cyanidin-3-O-(6''-acetyl-glucoside)	2.13	-	-
17. Malvidin-3-O-(6''-acetyl-galactoside)	9.91	-	-
18. Petunidin-3-O-(6''-acetyl-glucoside)	5.26	-	-
19. Malvidin-3-O-(6''-acetyl-glucoside)	15.2	-	-
Total ANC	206	382	25.1

'-', not detected
[a]Post-C18 extracts
[b]Adapted from Table 2 of Strathearn, K. E. et al., *Brain Res* **2014,** 1555, 60-77
[c]ANC concentrations were estimated by HPLC as the cyanidin-3-O-glucoside equivalent.

Supplementary Figure S1

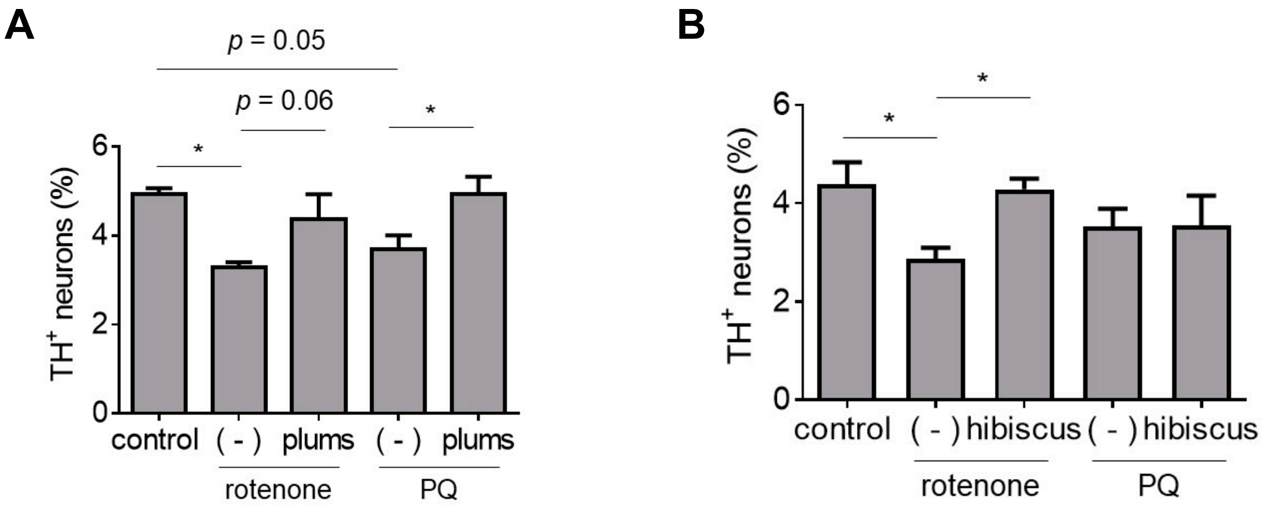

Supplementary Figure S1. Comparison of protective activities of polyphenol-rich extracts against neurotoxicity elicited by rotenone or PQ. Primary midbrain cultures incubated in the absence or presence of extract prepared from plums (A) or hibiscus (B) for 72 h were exposed to rotenone (25 nM) or PQ (2.5 μM) in the absence or presence of extract for 24 h. Control cells were incubated in the absence of rotenone, PQ, or extract. The cells were stained with antibodies specific for MAP2 and TH and scored for relative dopaminergic cell viability. The data are presented as the mean ± SEM; n = 2 (A) or n = 3 (B), *p<0.05, square root transformation, one-way ANOVA with Tukey's multiple comparisons post hoc test.

Supplementary Figure S2

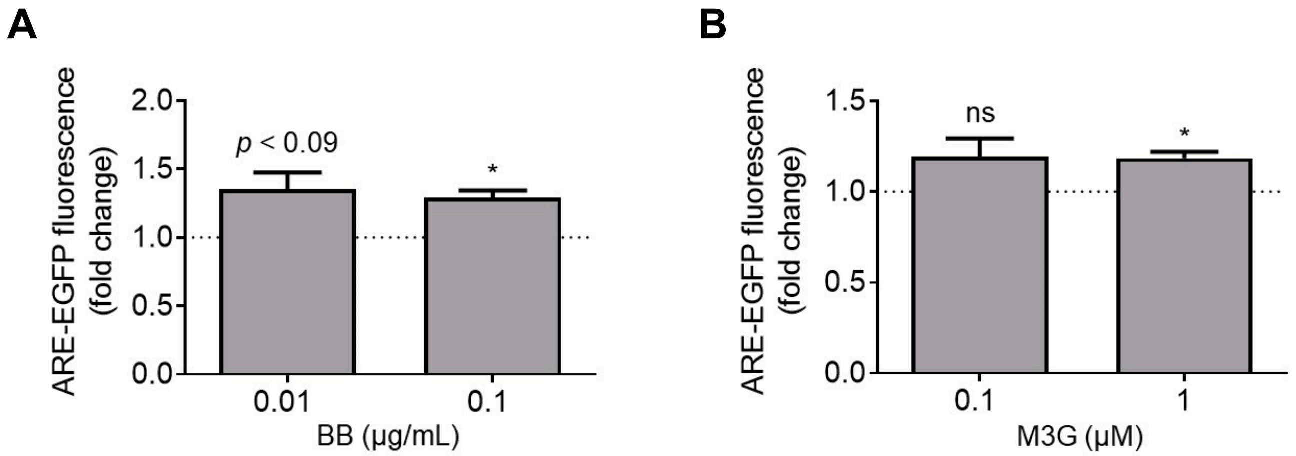

Supplementary Figure S2. Evidence of an increase in Nrf2 transcriptional activity in human iPSC-derived astrocytes treated with BB extract or M3G. iCell astrocytes transduced with an ARE-EGFP reporter adenovirus for 48 h were incubated in the absence or presence of BB extract (A) or M3G (B) for 24 h. Control astrocytes were transduced with the reporter virus and incubated in the absence of extract or compound. The cells were imaged to determine the intracellular EGFP fluorescence intensity. The data are presented as the mean ± SEM; $n = 3$; *$p<0.05$ versus a predicted ratio of 1, log transformation followed by one-sample t-test (ns, not significant).

Supplementary Figure S3

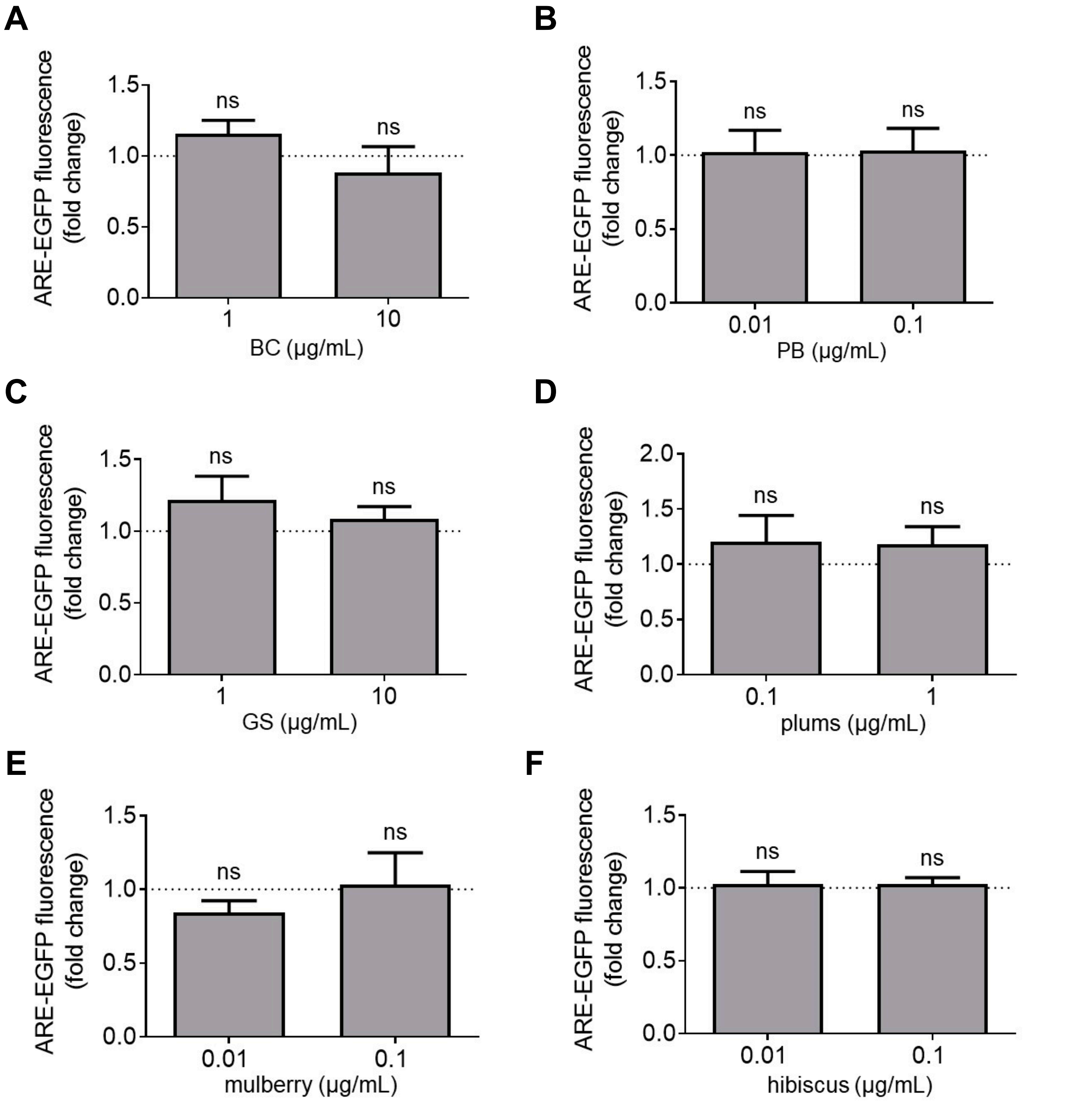

Supplementary Figure S3. A subset of botanical extracts fail to activate Nrf2. Primary cortical astrocytes transduced with an ARE-EGFP reporter adenovirus for 48 h were incubated in the absence or presence of extract prepared from BC (A), PB (B), GS (C), plums (D), mulberry (E), or hibiscus (F) for 24 h. Control astrocytes were transduced with the reporter virus and incubated in the absence of extract. The cells were imaged to determine the intracellular EGFP fluorescence intensity. The data are presented as the mean ± SEM; n = 3 (B, E, and F), n = 4 (A), or n = 6 (C and D) (ns, not significant).

Supplementary Figure S4

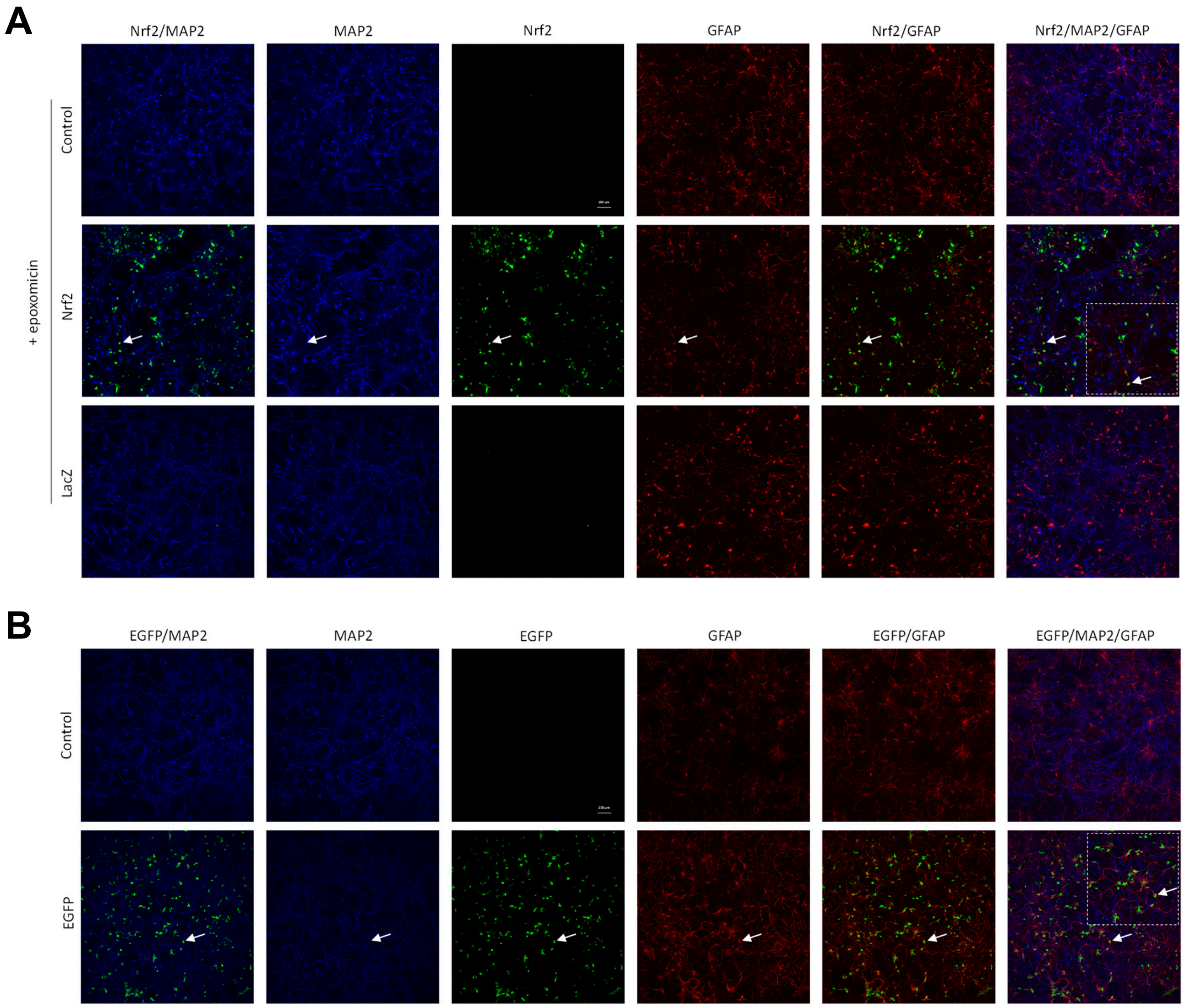

Supplementary Figure S4. Gene expression in mixed midbrain cultures transduced with Ad-CMV adenoviral vectors is restricted to astrocytes. (A) Primary midbrain cultures were incubated in the absence ('Control', top) or presence of adenovirus encoding Nrf2 (middle) or LacZ (bottom) under the control of the CMV promoter (MOI = 5 or 10, respectively) for 72 h and then treated with epoxomicin (50 nM) for 24 h to promote the accumulation of the labile Nrf2 protein. The cells were stained for Nrf2 (green), MAP2 (blue), and GFAP (red) and analyzed via confocal microscopy to determine the cell type-specificity of Nrf2 expression. (B) Primary midbrain cultures were incubated in the absence ('Control', top) or presence (bottom) of adenovirus encoding EGFP under the control of the CMV promoter (MOI = 10). The cells were stained for MAP2 (blue) and GFAP (red) and analyzed via confocal microscopy to determine the cell type-specificity of EGFP expression. In (A) and (B), each row consists of single-channel or merged (2- or 3-channel) images for one field of view, where the 3-channel merged image of cultures transduced with virus encoding Nrf2 (A) or EGFP (B) includes a 2.5x zoomed inset. Nrf2 and EGFP were found to be expressed in $GFAP^+$ astrocytes, but not in $MAP2^+$ neurons. Arrows in (A) and (B) point to an astrocyte expressing Nrf2 or EGFP, respectively, at the same position across all panels in each row. The absence of Nrf2 immunoreactivity in cultures transduced with LacZ virus (A, bottom) confirms that the Nrf2 signal observed in cultures treated with Nrf2 virus (A, middle) corresponds to the ectopically expressed protein rather than endogenous Nrf2 up-regulated as part of a stress response. Scale bar (A, B): 100 µm.
